# Longitudinal Associations between Emotion Regulation and Adiposity in Late Adolescence: Indirect Effects through Eating Behaviors

**DOI:** 10.3390/nu11030517

**Published:** 2019-02-28

**Authors:** Lenka H. Shriver, Jessica M. Dollar, Meg Lawless, Susan D. Calkins, Susan P. Keane, Lilly Shanahan, Laurie Wideman

**Affiliations:** 1 Department of Nutrition, University of North Carolina at Greensboro (UNCG), Greensboro, NC 27412, USA; m_lawles@uncg.edu; 2 Department of Human Development and Family Studies, University of North Carolina at Greensboro (UNCG), Greensboro, NC 27412, USA; jmdollar@uncg.edu (J.M.D.); sdcalkins@uncg.edu (S.D.C.); 3 Department of Psychology, University of North Carolina at Greensboro (UNCG), Greensboro, NC 27412, USA; spkeane@uncg.edu; 4 Jacobs Center for Productive Youth Development, University of Zurich, 8050 Zurich, Switzerland; lilly.shanahan@uzh.ch; 5 Department of Kinesiology, University of North Carolina at Greensboro (UNCG), Greensboro, NC 27412, USA; l_wideman@uncg.edu

**Keywords:** emotion regulation, obesity, emotional eating, adolescence, adiposity

## Abstract

The prevalence of obesity among U.S. youth continues to increase, with many adolescents engaging in unhealthy eating behaviors. Increasingly, research points to the role of self-regulation in obesity development, yet existing work has largely focused on young children and/or clinical adult populations. This multi-method longitudinal study (*N* = 153) utilized a path analysis to delineate links between emotion regulation (age 15), emotional eating and dietary restraint (age 16), and adiposity (% body fat) using a BodPod for body composition assessment (age 19). Emotion regulation was negatively associated with emotional eating (*β* = −0.30, *p* < 0.001) and positively associated with dietary restraint (*β* = 0.15, *p* < 0.05) at age 16, but was not associated with age 19 adiposity (*β* = −0.01, *p* = ns). Emotional eating was positively associated with adiposity (*β* = 0.24, *p* < 0.01). Indirect effects suggested that emotional eating, but not dietary restraint, at age 16 serves as a mechanism that helps explain the associations between emotion regulation and adiposity four years later. Results from this study suggest that both emotion regulation and emotional eating represent promising targets for that should be included in future interventions aimed at preventing adolescent obesity.

## 1. Introduction

The obesity epidemic among American youth is alarming, with 41.5% of 16–19 years old adolescents being overweight or obese [1]. The negative health consequences of obesity are well established, ranging from increased risk of diabetes and cardiovascular disease to poor psychological outcomes [2,3]. While obesity develops over time as a result of positive energy balance, caused by excessive dietary energy intake, inadequate energy expenditure, or a combination of both [4,5], it is well recognized that a complex set of physiological, environmental, and psychological factors are also involved in this process [3].

Dietary energy intake is controlled by the homeostatic system that is based on basic physiological needs for energy, but also by the hedonic pathway that triggers food intake regardless of energy stores [6]. Hedonic eating (i.e., reward-based eating) is recognized to have an increasingly important role in obesity development in recent decades, as a greater proportion of the population live in a so-called “obesogenic environment”, with widespread availability of high energy-dense foods [7,8]. At the same time, a growing body of work points to links between an individual’s self-regulatory abilities and obesity development [9,10]. The precise mechanisms that link self-regulation with obesity risk are not well defined, largely due to the reliance on cross-sectional studies that do not capture specific self-regulatory processes in relation to obesity risk. Furthermore, differences in the type (i.e., emotion regulation, inhibitory control) and measurement technique employed across studies of self-regulation have made it difficult to discern how and why self-regulation is associated with obesity risk [11]. 

One dimension of self-regulation, emotion regulation, is defined as those behaviors, skills, and strategies—whether conscious or unconscious, automatic or effortful—that serve to modulate, inhibit, and enhance emotional experiences and expressions [12], and it represents an important target of the investigation. Toddlers and preschoolers with better emotion regulation skills have lower odds of becoming overweight or obese compared to their peers with poor emotion regulation [13,14]. Despite dramatic growth in the development of skills to regulate emotional arousal in infancy and early childhood [15], biological and behavioral aspects of emotion regulation continue to be refined into late childhood and early adolescence. In fact, a meta-analysis by Mannen et al. [16] found associations between depression, which is marked by deficits in emotion regulation [17], and obesity in adolescents; depressed teens had a 70% increased risk of being obese compared to their non-depressed peers. Additional work is needed to establish the association between emotion regulation not tied explicitly to depressive symptoms and obesity in community samples of children and adolescents.

In addition to emotion regulation emerging as a potential target for improving nutrition-related outcomes, it is well established that a wide range of food-related behaviors influence dietary intake, with some eating behaviors being implicated in obesity development more than others [18,19]. Emotional eating is an eating behavior referring to the use of food as a coping strategy for negative affect and/or stressful life events, and is considered an atypical response to negative emotions [20]. While a typical physiological response to stress or negative feelings encompasses a decreased appetite [21], emotional eaters tend to turn to food to alleviate negative mood and unpleasant feelings [22]. Emotional eating has been strongly linked with binge eating and loss of control over eating [23,24]. The direct associations between emotional eating and obesity have been clearly demonstrated in previous research. Emotional eaters tend to consume sweets, salty foods and other high energy-dense foods, which contributes to excessive energy intake and increased obesity risk over time [18,25,26]. Although emotional eating has been primarily investigated in adult samples or those with disordered eating [19,24,27,28,29], research suggests that up to 63% of children and adolescents engage in emotional eating [26,30]. Thus, it is imperative to develop a better understanding of how emotional eating functions in relation to obesity risk in this vulnerable age group. 

Another aspect of eating that is important to consider in obesity risk is dietary restraint. Restraint is defined as the restriction of food intake, via cognitive effort, for weight control purposes [31,32]. However, restraint may also represent a risk factor for eating pathology development, such as anorexia or binge eating disorder [33]. Thus, restraint, as it relates to eating less than desired, has been investigated in both obesity and eating disorder literature [28,34]. To date, a straightforward association between dietary restraint and obesity risk has not been confirmed as some studies found positive [35,36,37] while other studies found no correlations between restraint and weight-related outcomes [33,38]. The inconsistent findings are likely due to differences in sample characteristics as the nature of the association is likely to vary by the individual’s current weight or dieting status [39]. Thus, further research is warranted to clarify the associations between restraint and obesity risk in non-clinical samples of children and adolescents. 

A growing body of work has established that a lack of appropriate emotion regulation skills is associated with increased risk of maladaptive eating behaviors. However, the association between poor emotion regulation and emotional eating has been, so far, investigated mostly in adult samples [40,41]. Only a few studies have examined these associations in children or adolescents [42,43]. Harrist and colleagues [42] reported that 1st graders who showed poor emotion regulation skills (i.e., increased reactivity to anger and worry) were more likely to engage in emotional eating between 2nd and 3rd grade. Similar findings were reported by Lu et al. [43], who found that emotion suppression was related to greater emotional eating in a sample of adolescents. 

These studies, however, examined emotion regulation and emotional eating alone or in relation to dietary patterns rather than obesity-related outcomes. Thus, further studies are warranted to clarify how emotion regulation and emotional eating function influence obesity risk in youth. Similarly, minimal research exists on the links between emotion regulation and dietary restraint in non-clinical samples of adolescents [22]. A study by Svaldi et al. [44] found that when participants with high restraint were asked to suppress their emotions after watching sadness-inducing films, they experienced an increased desire to overeat from baseline to post-test compared to low restrainers, who did not experience any changes. However, the study was limited by small sample size and only included adult females. Additional research is warranted to better explain associations between poor emotion regulation, dietary restraint, and the potential impact of these links on obesity-related outcomes. 

In summary, adolescents in the U.S. suffer from high rates of obesity, eat a poor diet, and often engage in unhealthy eating behaviors [1,30,45]. Adolescence is a developmental period when (1) parental controls decrease, and youth gain more independence, (2) opportunities for unhealthy behaviors, including an increase in unhealthy eating, and (3) long-term food-related behaviors are established. Therefore, delineating the links between emotion regulation, eating behaviors and obesity in adolescence using a longitudinal design is a high research priority [22,37]. 

We hypothesize that engagement in certain types of eating behaviors in adolescence is one mechanism by which emotion regulation and obesity are linked [31,46]. Specifically, we hypothesize that adolescents with poor emotion regulation will be more susceptible to using maladaptive eating behaviors (e.g., as a response to, and way to cope with, distress or negative feelings [47,48]; in turn, adolescents who engage in dysregulated eating behaviors will be at a greater risk of becoming overweight or obese. The current study employed a multi-method longitudinal study to: (1) assess whether adolescent emotion regulation at age 15 was associated with adolescent cognitive restraint and emotional eating at age 16 and adiposity at age 19; (2) examine whether dietary restraint and emotional eating at age 16 were associated with adiposity at age 19; and (3) examine if adolescent cognitive restraint and emotional eating at age 16 serve as mechanisms through which emotion regulation at age 15 was associated with adiposity four years later.

## 2. Materials and Methods 

### 2.1. Study Design and Participants 

This study utilized data from three cohorts of children who have been part of an ongoing longitudinal study of social and emotional development. Four hundred and forty-seven participants were initially recruited for the RIGHT Track study at two years of age through childcare centers, the County Health Department, and the local Women, Infants, and Children program. Additional details about the original sample recruitment and the adolescent health assessments that are part of the follow-up RIGHT Track Health study may be found elsewhere [49,50]. 

### 2.2. Procedures and Measures

The study was approved by the University of North Carolina Greensboro Institutional Review Board (#11-0360; PI Wideman and #09-0427; PI Calkins). Participants and their mothers participated in an ongoing longitudinal study beginning at age 2. The current analyses include data collected when children were 15, 16, and 19 years of age. During each visit, participants completed surveys, in addition to taking part in a variety of anthropometric, physiological, and metabolic assessments. If the participant could not complete a laboratory visit, survey packets were mailed to his/her home. Consent and assent processes were implemented so parents could provide permission for participation when the child was less than 18 years old. Prior to the 19-year laboratory visit, participants were asked to refrain from vigorous exercise and alcohol consumption for 24-h and to avoid eating and cigarette smoking for 2 h before their scheduled appointment time. The measures relevant to the current study are described below. 

#### 2.2.1. Emotion Regulation

At the 15-year laboratory visit, emotion regulation was assessed through an adolescent report on the Emotion Regulation Checklist for Adolescents (ERCA) [51]. The adolescent version of the ERCA is a 27-item scale (α = 0.86) that was adapted from the original parent-report 24-item measure to assess the adolescent’s emotion regulation abilities. The ERCA has been correlated with the Child Behavior Checklist and observer ratings of children’s emotion regulation in unstructured play activities, thus demonstrating high construct validity [52]. Participants were asked to answer each item using a scale from 1 to 5 (1 = *Never* to 5 = *Almost Always*). 

#### 2.2.2. Emotional Eating and Dietary Restraint 

As part of the 16-year visit, adolescents reported on their emotional eating and restraint on the Three-Factor Eating Questionnaire (TFEQ) [53]. The original TFEQ questionnaire has three general subscales (i.e., disinhibition, dietary restraint, and hunger), which was validated for ages 12 and up and has been shown to have good reliability [53]. Participants answered items using a true/false format or Likert-type response options, with specific items being reversed coded prior to analyses following previously published scoring procedures [53]. The emotional eating subscale, a specific dimension of disinhibition examined in the current study (3 items; α = 0.80) measured participants’ eating behavior in relation to negative emotions (e.g., “When I feel sad, I…”) and has demonstrated reliability and validity [54]. The restraint subscale (20 items; α = 0.82) assessed the participant’s intent to control food intake for weight management purposes (e.g., “I deliberately take small helpings as a means of controlling my weight”). Scores for both subscales were calculated by creating a mean of the responses, with higher scores indicating a greater level of the eating behavior. Although hunger was not a target construct in our model, in preliminary models, we examined associations between the TEFQ subscales. As expected, emotional eating was associated with dietary restraint (*r* = 0.17; *p* < 0.01) and with hunger (*r* = 0.36; *p* < 0.001). There was no significant association between dietary restraint and hunger (*r* = 0.01; *p* = 0.88).

#### 2.2.3. Adiposity

Given the well-established limitations of using body mass index (BMI)-for-age as a measure of adiposity [55], body fat percentage was utilized as the measure of adiposity in the current study. At age 19, adiposity (% body fat) of participants was assessed using air displacement plethysmography technique for body composition assessment via BodPod (Cosmed USA Inc., Concord, CA, USA) during a lab visit. The body composition assessment using the BodPod method has been shown to be a reliable and valid method for body density assessment across different populations, including children and adults [56]. Lean body mass and fat mass were estimated using age and race appropriate algorithms built into the BodPod; the current study employed percent body fat as the measure of adiposity. 

Prior to the body composition assessment, height and weight were measured using standard procedures. Height was measured using a wall-mounted stadiometer, and weight was measured using a balance beam. For descriptive purposes in the current study, the BMI-for-age percentiles at age 15 were used to categorize participants into four weight status categories (e.g., BMI-for-age percentile cutoff values: less than 5th percentile = underweight; 5th to less than the 85th percentile = healthy weight; 85th to less than 95th percentile = overweight; equal or greater than the 95th percentile = obese) in our sample [57]. 

#### 2.2.4. Statistical Analysis

Mplus (Version 7; [58]) was used to conduct a path analysis to examine the associations between adolescents’ self-reported emotion regulation at age 15, dietary restraint and emotional eating behaviors at age 16, and adiposity (percent body fat) at age 19. Full Information Maximum Likelihood (FIML) was used to handle missing data. Model fit was assessed by examining the comparative fit index (CFI) [59], the Tucker-Lewis index (TLI) [60], the standardized root mean square residual (SRMR), and the root mean square error of approximation (RMSEA) [61]. Values close to or greater than 0.95 indicate good model fit for the CFI, values less than 0.06 indicate good model fit for RMSEA, and values less than or equal to 0.08 indicate good model fit for SRMR [62]. A bias-corrected bootstrapping procedure (10,000 draws) was used to test the indirect effect of adolescents’ emotion regulation at age 15 on adiposity four years later, through adolescents’ restraint and emotional eating at age 16. This approach has been shown to generate the most accurate confidence intervals for indirect effects, reducing Type 1 error rates and increasing power over other similar tests [63].

## 3. Results

Data from a total of 153 participants were utilized in the current study. The sample was 56% of females. Participants were economically diverse based on Hollingshead (1975) scores at the 15-year assessment, with a range from 13.5 to 66.0 (*M* = 45.61, SD = 13.03), thus representing families from each level of social strata typically captured by this scale. The sample was diverse; 64.1% of the adolescents were European American, 29.9% African American, 3.6% biracial, and 2.4% identified as other race/ethnicity. Additional descriptive statistics and correlations for the main study variables are presented in Table 1. In our sample, 68.8% of participants fell into the normal/healthy weight category, 15.6% of participants were within the overweight category, and 15.6% of participants were obese. Within the normal/healthy weight category, emotional eating was significantly correlated with restraint (*r* = 0.34, *p* < 0.01). Emotional eating was not significantly correlated with restraint among overweight/obese participants (*r* = −0.11, *p* = 0.53).

Adolescents’ dietary restraint and emotional eating were significantly positively associated with one another. Given that adolescent sex was significantly associated with self-report of restraint, and percent body fat, and that SES (Socioeconomic Status) was significantly correlated with self-report of emotion regulation, sex and SES were included as covariates in the model.

**Figure 1:**
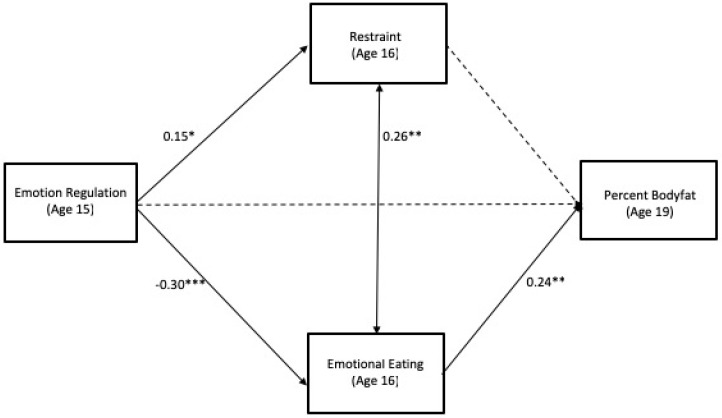
Standardized Estimates and Model Fit. Model Fit: *χ*^2^ (14, *N* = 167) = 96.84, *p* = 0.00, CFI = 0.95, RMSEA =0.05 [CI = 0.01, 0.15]. Note: Sex, SES were covariates; Non-significant paths are shown as dashed lines and significant paths are shown as solid lines. * = *p* < 0.05; ** = *p* < 0.01; *** = *p* < 0.001.

The hypothesized model was a good fit to the data, *χ*^2^ (14, *N* = 167) = 96.84 *p* = 0.00, CFI = 0.95, RMSEA = 0.05 [CI = 0.01, 0.15] (standardized coefficients are presented in Figure 1). The first aim of the study was to assess whether adolescent emotion regulation at age 15 was associated with adolescent dietary restraint and emotional eating at age 16 and percent body fat at age 19. Results indicated that self-reported emotion regulation at age 15 was negatively associated with emotional eating (*β* = −0.30, *p* < 0.001, CI [−0.44, −0.13]) and positively associated with restraint (*β* = 0.15, *p* < 0.05, CI [0.07, 0.35]) at age 16. This suggests that higher levels of emotion regulation at age 15 were associated with greater dietary restraint and lower emotional eating at age 16. Age 15 emotion regulation was not significantly associated with age 19 adiposity (*β* = −0.01, *p* = 0.90, CI [−0.22, 0.21]).

The second aim of the study was to examine whether dietary restraint and emotional eating at age 16 each were associated with percent body fat at age 19. Results indicated that emotional eating was positively associated with percent body fat (*β* = 0.24, *p* < 0.01, CI [0.01, 0.47]), suggesting that adolescents who engage in more emotional eating are more likely to have an increased percent body fat four years later. The association between restraint and percent body fat was non-significant (*β* = 0.13, *p* = 0.15, CI [−0.05, 0.30]). 

The final aim was to examine whether adolescent dietary restraint and emotional eating at age 16 serve as mechanisms through which self-reported emotion regulation at age 15 was associated with percent body fat four years later. The indirect effect from emotion regulation to percent body fat was significant via adolescent emotional eating (see Table 2 for unstandardized estimates of indirect effects). This indicates that adolescent emotion regulation was associated with percent body fat four years later through its association with adolescents’ engagement in emotional eating behaviors. The indirect effect of emotion regulation on percent body fat was non-significant via adolescent restraint. 

## 4. Discussion

The aim of the current study was to examine the longitudinal associations between emotion regulation, eating behaviors, and adiposity among adolescents. Despite some reports suggesting that obesity rates have stabilized in certain age groups [64], a recent study indicates that there has been a sharp increase in the prevalence of overweight and obesity in adolescents between 1999 and 2016 [1]. The current study advances the limited understanding of obesity-related processes by: (a) considering the role of emotion regulation and eating behaviors in adolescent obesity risk development, and (b) utilizing body fat percentage as a measure of adiposity rather than BMI, which has known limitations [55].

To date, the effectiveness of existing nutrition interventions for children and adolescents is limited with only small and/or short-lasting effects [65,66]. The role of self-regulation in obesity development has received a lot of attention in recent years, and self-regulatory abilities in early life have been found to predict weight outcomes later in childhood [9,13,46]. While different aspects of self-regulation may influence health behaviors across the lifespan (i.e., executive functioning, inhibitory control) [9], poor emotion regulation, in particular, has been linked to a wide range of negative nutrition-related outcomes in adults, ranging from poor diet quality to disordered eating [46]. Research investigating the role of emotion regulation in relation to obesity risk, however, has focused mostly on infants or children [13,14,43], largely because early childhood is a time period for a dramatic increases in self-regulation skills [15,49]. However, emotion regulation skills continue to develop and adolescents may have an increasing opportunity to regulate their own emotions and eating behaviors as parental controls decrease. 

The work presented here contributes significantly to the limited knowledge of how emotion regulation might influence obesity risk during adolescence by considering the direct and indirect pathways of emotion regulation in adolescence to subsequent adiposity four years later. Emotion regulation in our sample of adolescents was related to both emotional eating and dietary restraint, such that greater emotion regulation at age 15 was associated with lowered emotional eating but greater dietary restraint at age 16. The fact that adolescents with better emotion regulation reported lower levels of emotional eating in our sample is consistent with previous findings in children and adults [41,42]. A study by Lu et al. [43] demonstrated a similar association in a sample of adolescents, where a higher level of emotional suppression was associated with greater emotional eating in boys and girls. We found a positive association between emotion regulation and restraint among adolescents in our sample. Dietary restraint has been heavily researched, primarily in clinical settings, using the restraint theory [67], which suggests that restraint may contribute to the disruption of satiety responsiveness, increase overeating and/or also contribute to disordered eating. Our finding is aligned with recent studies that point to the positive role that restraint may play in healthy eating and successful weight management [39]. Restraint might be a necessary skill in order to successfully maintain energy balance in the current obesogenic environment [33]. Our findings highlight the need to consider restraint as a positive construct in light of the self-regulation theories [39]. 

Emotion regulation was not directly associated with adiposity in late adolescence in our sample. Although a few studies with young children have shown direct effects of emotion regulation skills on weight/obesity outcomes, our finding is not surprising given that obesity development is a complex process influenced by a variety of physiological, psychological, and social factors over time [3]. We did, however, expect, and find, that greater emotional eating would be linked with higher adiposity because emotional eating has been associated repeatedly with higher obesity risk, binge eating, and consumption of energy-dense foods [25,26]. Emotional eating has been examined in relation to dietary or weight outcomes among teenagers in recent research [43,68]. Emotional eaters tend to use “junk” food to remedy unpleasant feelings, which contribute to positive energy balance over time [5]. While interventions addressing emotional eating have been developed, most have focused on adults, females only, overweight/obese individuals, and/or those with disordered eating [68]. Our findings suggest that a focus on lowering emotional eating in adolescence could be of great importance in future preventive intervention efforts. 

Adolescent dietary restraint was not related to later adiposity in our sample. Research shows that the effect of restraint on weight or dietary outcomes may depend on the individual’s weight status or the degree of perceived restraint [33,69]. For instance, individuals with high levels of restraint might experience weight gain over time despite their high perceived cognitive restraint, due to exceeding their energy needs over time [33]. The outcomes of restraint may also vary depending on whether the individual engages in a chronic “flexible” restraint or whether she/he is in an acute phase as a response to recent weight gain which might trigger a more “rigid” restraint [33,69]. High restrainers might be more vulnerable to a variety of factors that trigger food intake after a period of restraint, such as external stimuli or negative emotions [39]. In our sample, adolescents with a higher restraint reported greater emotional eating and also a higher level of hunger. Although examination of eating behaviors by weight status was not the primary aim of the current study, in preliminary analyses, we found that these associations were only true for normal/healthy weight adolescents. Dietary restraint was not associated with emotional eating or hunger among overweight/obese participants in our sample. Previous research suggests that general disinhibition tends to correlate with restraint in overweight/obese individuals (suggesting “breaking of the diet,”); however, our study focused on the specific emotional dimension of disinhibition, which likely explains the lack of significant correlation found among overweight/obese individuals in our sample. As suggested by Schaumberg et al. [33], dietary restraint assessment is limited by the available measures and most studies have not been able to capture the gap between perceived restraint and actual energy restriction. Improved research tools for the assessment of dietary restraint are warranted in order to investigate its role in light of the self-regulation model proposed by Schaumberg et al. [33].

Finally, the indirect effect of emotion regulation at age 15 to adiposity at age 19 through emotional eating, but not dietary restraint, at age 16 was significant. Our findings suggest that adolescent emotion regulation might be particularly salient in pathways towards obesity risk and that one pathway by which adolescents, who do not have strong emotion regulation skills, are at risk for obesity is through increased likelihood of engaging in emotional eating [42,43]. As emotional eating is associated with greater intake of high energy-dense foods [26,43], lack of emotion regulation skills may indirectly contribute to positive energy balance. Given the current high rates of obesity among adolescents and their emotional vulnerability as they face many physiological, psychological, and social transitions during this developmental stage [70], our findings are notable and greatly add to the literature. Numerous childhood obesity prevention programs have been developed, yet a vast majority have failed to show practically relevant and/or long-lasting positive effects [71]. To our knowledge, there are currently no programs that target both emotion regulation and emotional eating as tools for helping adolescents in community-based settings establish healthy eating behaviors and prevent the use of food as a response to negative feelings. The longitudinal findings of the current study fill an important gap and point to the importance of approaching obesity prevention from a multidisciplinary angle and addressing non-nutrition factors, such as emotion regulation, in community settings. 

The present study has multiple, notable strengths. First, the study utilized data from a relatively large community sample of adolescents. The richness of the data is unique and allowed for an in-depth examination of the associations between the target constructs. Second, the longitudinal design with multiple data collection time points between ages 15 and 19 allowed us to examine the direction of effects between emotion regulation at age 15, eating behaviors at age 16, and adiposity at age 19. Third, the main outcome of the study was percent body fat that was assessed by estimating body composition using the BodPod technique—a more accurate measure of adiposity than BMI. The use of BMI as a measure of adiposity has well-known limitations, including having limited sensitivity to identify individuals with excess body fat [55]. Finally, the current study advances the existing literature by moving beyond describing simple associations but rather identifying mechanisms that explain the links between emotion regulation, eating behaviors, and adiposity in the target population. 

The study also had some limitations that must be noted. The assessment of emotion regulation and eating behaviors was based on self-report and thus were subject to a certain level of bias. However, adolescent self-report of emotion regulation is, in fact, a strong measure of self-regulation in this age group, while direct observations are the best method for early childhood. While all measures utilized in the study were validated and used in previous studies with similar sample characteristics, future work should also include biological and/or observational measures of emotion regulation to confirm the associations found in the current study. Also, a further examination of the construct of hunger, using a combination of perceived and physiological markers of hunger, in relation to eating behaviors and obesity risk among adolescents is warranted. Lastly, parental behaviors and/or beliefs related to food were not assessed in the current study but should be considered in future research in this age group.

## 5. Conclusions

Emotional eating in mid-adolescence is not only related to higher adiposity, but it also functions as the mechanism through which adolescent emotion regulation impacts adiposity in late adolescence. Both emotion regulation and emotional eating represent promising targets for effectively preventing and/or reducing adolescent obesity in future interventions. 

## Figures and Tables

**Table 1:** Characteristics of the Adolescent Sample and Correlations among Model Variables.

	1	2	3	4	5	6
1. Sex	--					
2. 15-year SES	−0.15	--				
3. 15-year ER	0.10	0.17 *	--			
4. 16-year Dietary Restraint	0.28 **	0.03	0.17 *	--		
5. 16-year Emotional Eating	0.06	0.05	−0.30 **	0.17 **	--	
6. 19-year Percent Body Fat	0.55 ***	−0.16	0.03	0.33 **	0.28 **	--
Mean	1.55	45.61	3.94	0.34	0.50	26.12
Minimum	1.00	13.00	2.56	0.05	0.00	2.80
Maximum	2.00	66.00	4.89	0.90	3.00	52.80
Standard Deviation	0.50	13.03	0.44	0.20	0.96	11.97

* *p* < 0.05, ** *p* < 0.01; *** *p* < 0.001; SES = Socioeconomic Status; ER = Emotion Regulation.

**Table 2:** Unstandardized Estimates of Indirect Effects, Standard Errors, and 95% Bias-Corrected Bootstrap Confidence Intervals.

			Confidence Intervals	
	Estimate	SE	Lower	Upper
ER(15yr) → Dietary Restraint(16yr) → Percent Body Fat(19yr)	0.55	0.46	−0.14	2.55
ER(15yr) → Emotional Eating(16yr) → Percent Body Fat(19yr)	−1.97 *	0.90	−4.67	−0.27

Note: ER = Emotion Regulation; * = *p* < 0.05.
